# Immunogenicity and Safety of Anti-SARS-CoV-2 mRNA Vaccines in a Cohort of Patients with Hereditary Angioedema

**DOI:** 10.3390/vaccines11020215

**Published:** 2023-01-18

**Authors:** Ilaria Mormile, Maria Celeste Gigliotti, Angelica Petraroli, Antonio Cocchiaro, Alessandro Furno, Francescopaolo Granata, Francesca Wanda Rossi, Giuseppe Portella, Amato de Paulis

**Affiliations:** 1 Department of Translational Medical Sciences, University of Naples Federico II, 80131 Naples, Italy; 2 Center for Basic and Clinical Immunology Research (CISI), University of Naples Federico II, 80131 Naples, Italy; 3 WAO Center of Excellence, 80131 Naples, Italy

**Keywords:** angioedema, hereditary angioedema, hereditary angioedema due to C1-esterase inhibitor deficiency, COVID-19, anti-SARS-CoV-2 mRNA vaccines

## Abstract

Many factors may trigger hereditary angioedema (HAE) attacks. This study aims to gain insights into the benefits and potential risks of COVID-19 vaccination in HAE patients, focusing particularly on the possibility of triggering attacks. We enrolled 31 patients with HAE undergoing two doses of the SARS-CoV-2 mRNA Comirnaty-BioNTech/Pfizer vaccine. To evaluate the possible influence of the vaccine on disease control and attack frequency, we administered the angioedema control test (AECT) 4-week version before (T0), 21 days after the first dose (T1), and between 21 and 28 days after the second dose (T2). Despite 5 patients (16.1%) experiencing attacks within 72 h of the first dose administration, no significant variation in attack frequency was observed before and after vaccination [F(2,60) = 0.123; *p* = 0.799]. In addition, patients reported higher AECT scores at T1 and T2 compared to T0 [F(2,44) = 6.541; *p* < 0.05; post hoc *p* < 0.05)], indicating that the disease was rather more controlled after vaccinations than in the previous period. All patients showed a positive serological response to the vaccine without significant differences from healthy controls (U = 162; *p* = 0.062). These observations suggest that the vaccine administration is safe and effective in HAE patients.

## 1. Introduction

Angioedema without wheals (AE) is a potentially life-threatening disease characterized by swelling of cutaneous and subcutaneous tissue due to increased vascular permeability caused by the increased release of vasoactive mediators such as bradykinin [1,2]. This rare disease is currently classified into hereditary (HAE) and acquired forms (AAE) [3,4,5]. Hereditary angioedema due to C1-esterase inhibitor deficiency (C1-INH-HAE) is caused by reduced synthesis (C1-INH-HAE type I) or the production of an unfunctional C1-INH protein (C1-INH-HAE type II) due to mutations in the C1-INH gene (SERPING1) [3]. Other HAE subtypes with normal C1-INH level (HAEnCI) have been identified, produced by mutations in other genes (i.e., factor 12 (FXII-HAE), plasminogen (PLG-HAE), angiopoietin-1 (ANGPT1-HAE), kininogen-1 (KNG1-HAE), myoferlin (HAE-Myoferlin), and HS3ST6 gene encoding for heparan sulfate glucosamine 3-O-sulfotransferase 6 (3-OST-6)) [6,7,8]. HAE patients show a broad clinical expression since the attacks may involve several anatomical districts, ranging from the skin to the gastrointestinal tract [9,10] and respiratory airways [11].

Vaccination is a pivotal tool to control the diffusion of the current Coronavirus Disease 2019 (COVID-19) pandemic and to decrease related complications. The novelty of the mRNA vaccines initially led to some patients’ diffidence and insecurity, especially in those with immune system disorders. Even though single severe reactions have occurred worldwide after mRNA-based COVID-19 vaccination, these types of reactions are considered rare [12,13,14]. An allergic workup using a screening questionnaire to estimate the probability of an allergic reaction to the COVID-19 mRNA vaccines in allergic people is recommended [13]. Because of its rarity and similar clinical presentation, HAE is often poorly recognized and confused with other allergic conditions [6,15]. Moreover, several factors may trigger HAE attacks, including emotional stress, physical trauma, and invasive medical procedures [6]. For these reasons, evaluating the safety of the new mRNA-based COVID-19 vaccine and its potential effects on HAE attack rate in a significant, homogenous cohort of HAE patients could be useful for increasing physicians’ awareness about this rare condition and better addressing the decision-making process about vaccination.

This study aims to gain insights into the benefits and potential risks of COVID-19 vaccination in HAE patients, mainly focusing on the possibility of triggering attacks.

## 2. Materials and Methods

### 2.1. Patients

Thirty-one patients diagnosed with HAE at the Campania Referral Center for Recurrent Angioedema, University of Naples Federico II, Naples, Italy, were enrolled in this prospective observational study. Inclusion criteria were a physician HAE diagnosis; a written informed consent signature; and available information about sex, demographic age and age at onset and diagnosis. We excluded patients with a positive history of COVID-19 infection.

Eighteen healthy age- and gender-matched subjects without previous COVID-19 infection (i.e., negative history of COVID-19 infection and anti-trimeric spike protein-specific IgG antibodies to SARS-CoV-2 (< 33.8 BAU/mL) at the time of enrollment) were chosen as controls. All enrolled participants received two doses of the SARS-CoV-2 mRNA Comirnaty^®^-BioNTech/Pfizer vaccine.

All consultations were performed in person, on an outpatient basis, at the Campania Referral Center for Recurrent Angioedema, University of Naples Federico II, Naples, Italy, 7 days before (T0), 21 days after the first dose (T1), and between 21 and 28 days after the second dose (T2). At the time of enrollment, information about comorbidities, medications, and routine laboratory investigations (i.e., complete blood count, liver function tests, kidney function tests, LDH, glucose, lipid panel) was also collected. During each follow-up visit, participants were asked about any vaccine-related adverse events through a standard questionnaire to gain insight into the safety and tolerability of the vaccines in HAE patients. This study was conducted in accordance with the ethical standards of the study center and with the 1964 Helsinki Declaration and its later amendments or comparable ethical standards. The Ethics Committee of the University Hospital Federico II of Naples, Italy, approved the study protocol. Prior to study participation, patients signed an informed consent form.

### 2.2. Disease Activity Assessment

#### 2.2.1. Angioedema Control Test

To evaluate the possible impact of the SARS-CoV-2 mRNA on disease control and attack frequency, we administered the angioedema control test (AECT) 4-week version [16] at T0, T1, and T2.

AECT is a self-administered 4-item patient-reported outcome measure for patients with recurrent AE. Scores between zero and four are assigned to every AECT answer. AECT may range from 0 to 16, with a higher score indicating a higher level of disease control (i.e., an AECT score < 10 points indicates poorly controlled recurrent angioedema, AECT score ≥ 10 indicates well-controlled angioedema). The questions cover the frequency of AE, its impact on quality of life (QoL), the level to which it troubled participants, and their ability to control it.

In addition, we asked the patients to provide the number of attacks experienced during the 21 days before each vaccine dose administration, during the 72 h following the first and the second dose, and during the 21 days after the second dose.

#### 2.2.2. The Hospital Anxiety and Depression Scale

The novelty of mRNA vaccines has caused some concern, particularly in patients affected by rare disorders. It is well known that anxiety and emotional distress may trigger attacks in patients with HAE. For these reasons, we used the hospital anxiety and depression scale (HADS) [17] to detect vaccine-related depression and anxiety in our patients. HADS is a self-rating scale composed of 14 items: 7 related to anxiety and 7 related to depression. Each item is scored from 0 to 3, so the score may vary between 0 and 21 for either anxiety or depression. Recommended cut-offs are 8 to 10 for doubtful cases and ≥11 for definite cases. We administered HADS at the first dose (T0) and the second dose (T1).

#### 2.2.3. Severity Score

The clinical severity of HAE was classified using the severity score proposed by Bygum et al. [18], ranging from 0 to 10, based on age at onset, attack site, and the need for long-term prophylaxis.

### 2.3. Blood Sample Collection

To study the short-term immunological effects of the vaccine in HAE patients, 29 out of 31 patients provided a blood sample to investigators at enrollment (T0) and during each follow-up visit (T1 and T2). Healthy controls provided a blood sample at T0 and at T2.

Chemiluminescence immunoassay (CLIA) was used to quantitatively determine anti-trimeric spike protein-specific IgG antibodies to SARS-CoV-2 (IgT), according to the manufacturer protocol (kit LIAISON Ctrl SARS-CoV-2 TrimericS IgG, DiaSorin S.*p*.A.). The assay reveals the presence of neutralizing IgG antibodies against SARS-CoV-2. The LIAISON^®^ SARS-CoV-2 TrimericS IgG assay measures between 4.81 and 2080 BAU/mL. Test results are stated as positive (≥33.8 BAU/mL) or negative (<3.8 BAU/mL), along with a numeric value for quantitative measurement.

### 2.4. Statistical Analysis

Data were summarized by descriptive analysis. Values were presented as frequency (number and percentage) and mean ± standard error of the mean. Repeated-measures 1-way ANOVA was used to analyze the number of attacks experienced during the 21 days before each vaccine dose administration and the 21 days after the second dose. Repeated-measures 1-way ANOVA was also used to analyze the dependent variable AECT with T0, T1, and T2 visits as a within-subject factor. We performed post hoc comparisons with dependent t-tests corrected with the Sidak procedure. Analysis of the dependent variable HADS was performed with the Wilcoxon test for the depression score and with a paired t-test for the anxiety score, comparing the two visits at T0 and T1. We performed the analysis of the dependent variables “anti-SARS-CoV-2-IgG levels” at T0 and T2 with the Mann–Whitney test comparing the two groups (“HAE patients” vs. “healthy controls”). We analyzed the dependent variable severity score with an unpaired t-test comparing the two groups of patients with or without attacks within 72 h of vaccination. Assumption of normality was performed with K-S and S-W tests. The assumption of homoscedasticity was performed with the Levene test. Alpha was 0.05. All analyses were performed with IBM SPSS Statistics for Mac, Version 28.0.1.0. 3.

## 3. Results

### 3.1. Demographics

Overall, 31 adult patients (9 males and 22 females) diagnosed with HAE (23 C1-INH-HAE with type I, 3 with type II, 3 with HAE-Myoferlin, and 2 patients with FXII-HAE) were enrolled in this study. Mean age was 45.3 ± 19 years (range 18–82) for C1-INH-HAE type 1, 57.7 ± 9.1 years (range 51–68) for C1-INH-HAE type 2, 49 ± 14.1 years (range 39–59) for FXII-HAE, and 29 ± 16.8 years (range 16–48) for MYOF-HAE. The mean time from diagnosis was 17.7 ± 14 for C1-INH-HAE type 1, 20.3 ± 16 for C1-INH-HAE type 2, 6 ± 1.4 for FXII-HAE, and 1 ± 0 for MYOF-HAE. Clinical features in our cohort are displayed in Table 1.

Attack frequency was investigated (Table 1). Information about on-demand treatment and long-term prophylaxis (LTP) was collected. Eleven out of twenty-four (45.8%) patients with C1-INH-HAE type I, and two out of seven patients with C1-INH-HAE type II (66.7%), were currently taking medication to prevent attacks on an ongoing basis. The most frequently used treatments for LTP were androgens (seven patients with C1-INH-HAE type I (63.3%) and two patients with C1-INH-HAE type II (100%); danazol and stanozolol) and C1-INH replacement products (four patients with C1-INH-HAE type I (36.4%); intravenous pdC1-INH concentrate). Thirteen out of thirty-one (41.9%) patients presented with the following comorbidities: hypertension (*n* = 3, 9.6%; treated with calcium channel blockers and beta-blockers), obesity (*n* = 3, 9.6%), type 2 diabetes mellitus (*n* = 2, 6.4%; treated with insulin therapy), thyroiditis (*n* = 2, 6.4%; treated with levothyroxine), allergic rhinitis (*n* = 2, 6.4%, in clinical remission), and chronic obstructive pulmonary disease (*n* = 1, 3.2%; treated with inhaled corticosteroids and bronchodilators). One patient was affected by coeliac disease in clinical remission since the beginning of the elimination diet that she started 20 years before this study. One patient was affected by psoriatic arthritis in monotherapy with an anti-TNF monoclonal antibody (golimumab). All the comorbidities in our cohort were in good clinical control with the therapies used. Routine laboratory investigations were within normal limits for all HAE patients.

### 3.2. Clinical Evaluation

The mean severity score [18] was 6.1 ± 2.5 and ranged from 0 to 10. In particular, in patients with type 1 C1INH-HAE the mean severity score was 7.1 ± 1.8 and it was 6.33 ± 1.5 in type 2 patients, whereas this score was lower in patients affected by FXII-HAE and MYOF-HAE (0.5 ± 0.7 and 3 ± 1 points, respectively). No significant difference was found between the severity scores of patients with or without attacks within the 72 h after vaccine administration [t(28) = 1.85; *p* = 0.075].

The average frequency of attacks was 1.8 ± 2 in the three weeks prior to vaccination (T0), 1.7 ± 4 at T1, and 1.9 ± 2 at T2 (21–28 days after the second dose vaccination). ANOVA conducted on the number of attacks did not reveal a significant difference between the T0, T1, and T2 [F(2,60) = 0.123; *p* = 0.799], demonstrating that the anti-SARS-CoV-2 vaccine did not impact the frequency of attacks.

**Figure 1:**
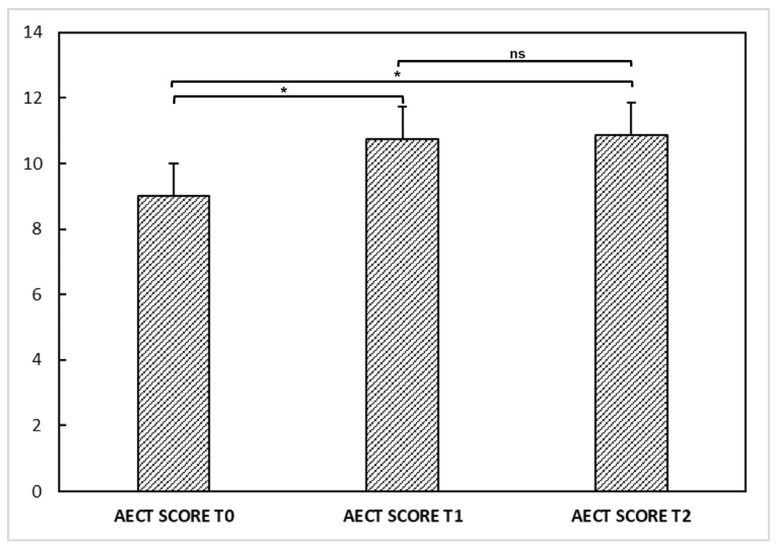
Angioedema control test (AECT) questionnaire at T0, T1, and T2 in our hereditary angioedema patients. ANOVA performed on AECT showed a significant difference (*) among the determinations at T0, T1, and T2 [F(2,44) = 6.541; *p* < 0.05]. Post hoc comparisons showed a higher AECT score at T1 (post hoc *p* < 0.05) and T2 (post hoc *p* < 0.05) compared to T0, suggesting that the disease was rather more controlled after vaccinations than in the previous period. No significant difference (ns) was observable between T1 and T2 (post hoc *p* = 0.994).

We also evaluated disease activity at different times through the AECT questionnaire. AECT was available for 23 out of 31 patients. The mean AECT score was 9 ± 3.5 points at T0, 10.7 ± 3.3 at T1, and 10.8 ± 3.2 at T2. ANOVA performed on AECT showed a significant difference among the three visits [F(2,44) = 6.541; *p* < 0.05]. Post hoc comparisons revealed a higher AECT score at T1 (post hoc *p* < 0.05) and T2 (post hoc *p* < 0.05) compared to T0, suggesting that the disease was rather more controlled after vaccinations than in the previous period. No significant difference was observable between T1 and T2 (post hoc *p* = 0.994) (Figure 1).

However, 5 out of 31 patients (16.1%) had an angioedema attack within 72 h of the first vaccination dose. Four out of five attacks were peripheral, while only one was abdominal. Three attacks were mild, so patients did not treat them, while two were moderate and were treated with rescue medication (one patient used icatibant while the other used pdC1INH). After the second vaccination dose, 6 out of 31 patients (19.3%) had an angioedema attack (3 abdominal and 3 peripheral). Four attacks had moderate intensity and were treated with pdC1INH, while two attacks were mild and not treated.

**Figure 2:**
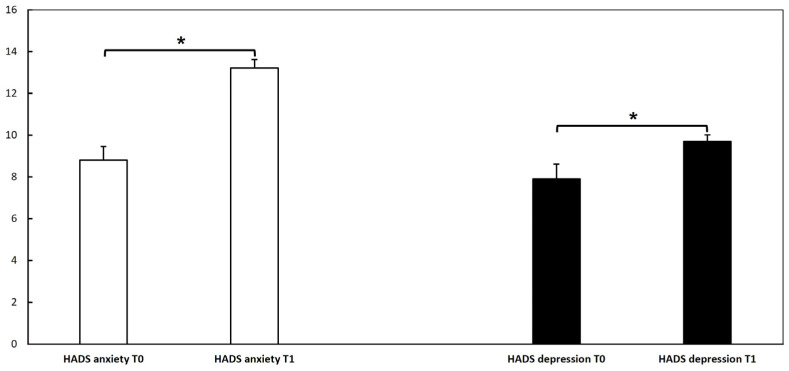
Hospital anxiety and depression scale (HADS) questionnaire at T0, T1, and T2 in our hereditary angioedema cohort. The analysis of dependent variable HADS for depression was performed with the Wilcoxon test, and for anxiety with a paired t-test; it revealed a significant difference (*) between T0 and T1 (*p* < 0.05), showing a higher score at T1 (at the second dose) compared to T0 (at the first dose). These results could suggest that vaccination may have affected patients’ anxiety status.

We assessed patients’ anxiety and depression at the first and second doses of vaccination through the HADS questionnaire. The mean HADS depression score at T0 was 7.9 ± 4.1, while the mean HADS anxiety score was 8.8 ± 3.6. At T1, the mean HADS depression score was 9.7 ± 1.8, whereas the HADS anxiety score was 13.2 ± 2.4. At T0, 9 patients had a total depression score ≥ 11, and 13 patients had a total anxiety score ≥ 11. At T1, 10 patients had a total depression score ≥ 11, while 27 patients had a total anxiety score ≥ 11. Analysis of the dependent variable HADS revealed a significant difference between the two visits (*p* < 0.05) for both anxiety and depression, showing a higher score at T1 compared to T0. These results could suggest that vaccination may have affected patients’ anxiety status with higher scores after the second dose than the first dose (Figure 2). Anxiety is a well-known trigger factor for HAE attacks, which may drop patients into a vicious circle, with further anxiety being a trigger factor for further attacks.

We also investigated whether patients had post-vaccination symptoms after the first and second doses of the vaccine, and compared them with data from a large Italian cohort recently reported by Monami et al. [19] (Table 2).

### 3.3. Anti-SARS-CoV-2 Vaccine Immunogenicity

**Figure 3:**
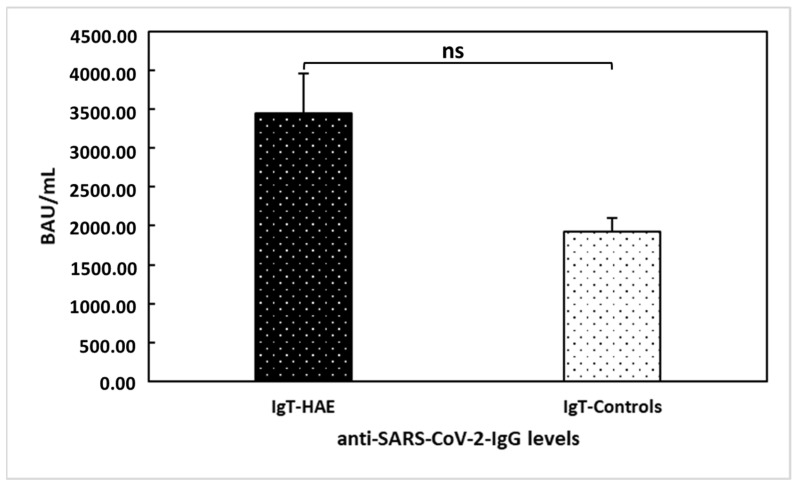
Comparison between hereditary angioedema (HAE) patients’ and healthy controls’ anti-trimeric spike protein-specific IgG antibodies to SARS-CoV−2 (IgT) levels at T2. No significant difference (ns) was found between the two groups of patients (U = 162; *p* = 0.062).

Our research group previously demonstrated [20] that HAE patients showed a significant serological response after a single dose of the vaccine (T1), with a further increase after the second dose (T2) (i.e., mean anti-SARS-CoV-2-IgG levels were 2.25 ± 4.26 at T0, 895.76 ± 1067.43 at T1, and 3443.97 ± 2753.72 at T2). The only patient treated with anti-TNF monoclonal antibody showed a positive serological response after the first dose of the vaccine, with an additional increase after the second dose (anti-SARS-CoV-2-IgG levels at T0 < 4.81 BAU/mL; T1: 124 BAU/mL; and T2: 686 BAU/mL). Herein, we compared anti-SARS-CoV-2-IgG levels between the 29 HAE patients providing blood samples and 18 healthy age- and gender-matched controls at T2 (Figure 3). No significant difference was found between the two groups (U = 162; *p* = 0.062).

## 4. Discussion

It has been postulated that patients with HAE are at increased risk for COVID-19 infection due to inherent dysregulation of the plasma kallikrein-kinin system [21]. However, there are highly complex interrelationships between SARS-CoV-2 and some of the molecules that play a significant role in HAE pathogenesis, including the contact and coagulation systems, angiotensin-converting enzyme-2 (ACE2), and bradykinin [22,23,24]. Indeed, the activation of bradykinin receptor 1 (B1R) and bradykinin receptor 2 (B2R) on endothelial cells in the lungs, which causes vascular alterations resulting in a localized kinin-dependent local lung angioedema, has been proposed as part of the pathogenetic mechanism involved in the development of pulmonary edema in COVID-19 [25]. In a recent research work [25], the authors hypothesized that the B2R-mediated inhibition of plasma kallikrein activity might improve early disease caused by COVID-19, possibly preventing acute respiratory distress syndrome (ARDS). In this view, some drugs currently used for treating acute angioedema attacks (e.g., the B2R antagonist icatibant) or for LTP (e.g., the plasma kallikrein inhibitor lanadelumab) may target the bradykinin-driven pulmonary edema observed in COVID-19 [24,25]. In addition, SARS-CoV-2 penetrates cells of the lungs and respiratory system by interacting with the viral glycosylated spike protein and ACE2, possibly causing a depletion of this enzyme [26] with consequent bradykinin accumulation, inducing vasodilation, lung injury, and inflammation [22].

Finally, complement is critical in the host’s immune response to viruses. Especially, the activation of complement C3 has been shown to exacerbate SARS-CoV-associated ARDS [27,28]. Moreover, Gralinski et al. [29] reported that C3-deficient mice infected with SARS-CoV showed lower cytokines and chemokines levels in both the lungs and sera, depletion of infiltrating neutrophils and monocytes in the lungs, and less respiratory dysfunction.

However, the studies performed on HAE attacks during the COVID-19 pandemic demonstrated increased attacks, with stress created by the fear of COVID-19 infection but no direct association with the infection itself [30,31,32].

HAE is a potentially life-threatening disorder associated with a significant emotional burden [33,34]. Indeed, this rare clinically heterogeneous condition is often accompanied by uncertainty due to the variability in the clinical phenotype, even in patients with the same genetic mutation, and the lack of reliable biomarkers able to predict the severity of the disease [35,36]. Several trigger factors may induce HAE attacks disrupting the vascular endothelium [37]. Short-term prophylaxis (STP) with pdC1INH is generally recommended before invasive medical procedures, but there are no specific guidelines about the management of intramuscular injection or vaccine administration in HAE patients [37,38]. Moreover, mRNA vaccines may frequently cause side effects such as fever, fatigue, and pain, which may trigger HAE attacks. In addition, RNA is a potent activator of the contact system, potentially further increasing the risk [38,39].

Emotional stress is a well-known trigger factor for HAE attacks [33], even though the underlying pathogenetic mechanisms behind this relationship are not elucidated. Some authors hypothesized the existence of a connection between autonomic nervous system dysfunction and the activation of the contact/complement system, suggesting a link between stress and bradykinin production [40]. A recent survey by Banerji and colleagues [41] showed a direct correlation between the frequency of attacks and the disease burden, showing that worse anxiety and depression (assessed by the HADS) were related to a higher attack frequency. As assessed in our HAE patient cohort by the HADS scale (Figure 2), vaccination may have affected patients’ anxiety status, with higher scores at the second dose of the vaccine than at the first dose. The difference between the two HADS determinations may be due to anticipatory anxiety related to the fear of side effects, since over half of our patients experienced at least one post-vaccination symptom after the first dose. However, despite this difference, the number of patients experiencing HAE attacks within the 72 h after vaccination was relatively low, with only a slight increase at the second dose (*n* = 6; 19.3%) compared to the first dose (*n* = 5; 16.1%). In addition, overall attack frequency and disease control were not significantly worsened by the vaccine. Indeed, no significant variation in attack frequency was observed before and after vaccination. Patients reported higher AECT scores at T1 and T2 (Figure 1), indicating that the disease was more controlled after vaccinations than in the previous period. These observations, taken together, suggest that the vaccine administration is safe in patients with HAE.

The current literature about COVID-19 vaccination in HAE patients is scarce. However, our results aligned with data from a large cohort of HAE patients from the Netherlands, investigating 63 patients with HAE who received at least one dose of different COVID-19 vaccines [38]. The authors reported 11 HAE attacks following the administration of 111 COVID-19 vaccines, 9 following first-dose administration. As in our cohort, all the attacks were of mild or moderate severity, and no patients developed laryngeal attacks or required hospital admission [38]. Most patients did not use STP before vaccine administration. Another study by Oztop et al. [32] analyzed the number and severity of attacks in 140 Turkish patients with HAE receiving two or three doses of the vaccine and reported that 18 patients (15.8%) experienced an HAE attack within 48 h of COVID-19 vaccination, but that the number and severity of the attacks were not globally increased. In addition, the authors reported that attacks after vaccination were mostly seen in patients with poorly controlled HAE assessed with the Bygum severity score [18]. On the contrary, we did not find a significant difference in the severity score of patients with or without attacks within 72 h of vaccination.

We also investigated whether patients reported adverse reactions after the first and second doses of the vaccine and compared them with data from a large Italian cohort recently reported by Monami et al. [19] (Table 2). Generally, the most frequently reported adverse events were reactions at the vaccination site (e.g., pain, swelling, itching, or redness on the injection site). In addition, in both cohorts, adverse events were more frequent, although not more severe, after the second dose. Differences in symptom frequencies are also probably due to the small sample examined in our study. Indeed, our study is subject to some limitations, including the single-center design and the small sample size. Larger cohorts of HAE patients should be examined to assess if differences exist in vaccine-related adverse reactions between HAE patients and the general population. However, in our cohort and in the other existing studies analyzing HAE patients undergoing COVID vaccination [32,38], no severe (e.g., hypersensitivity reaction) or fatal vaccine-related adverse effects occurred.

In conclusion, our findings suggest HAE patients can be safely vaccinated against COVID-19 without SPT, providing the availability of an effective on-demand attack treatment.

## Figures and Tables

**Table 1:** Characteristics of HAE patients in our cohort (*n* = 31).

Characteristic	C1-INH-HAE Type 1	C1-INH-HAE Type 2	FXII-HAE	MYOF-HAE
**No. of patients** **(%)**	23 (74.2%)	3 (9.7 %)	2 (6.4 %)	3 (9.7%)
**Female gender = 22 (71%)** **(*n*, %)**	16 (69.6%)	1 (33.3%)	2 (100%)	3 (100%)
**Caucasian ethnicity** **(*n*, %)**	23 (100%)	3 (100%)	2 (100%)	3 (100%)
**Age, years** **(mean, range)**	45.3 ± 19 (18–82)	57.7 ± 9.1 (51–68)	49 ± 14.1 (39–59)	29 ± 16.8 (16–48)
**Time from diagnosis** **(years; mean ± SD)**	17.7 ± 14	20.3 ± 16	6 ± 1.4	1 ± 0
**Attack frequency** **(mean/year)**	17/year	23/year	0/year *	1/year
**Severity score**	7.04	6.3	0	3
**LTP** **(*n*, %)**	11 (54.6%)	2 (66.7 %)	-	-
**Attenuated androgens** **(*n*, %)**	6 (45.4%)	2 (100%)	-	-
**pdC1-INH** **(*n*, %)**	5 (36.4%)	-	-	-
**Tranexamic acid** **(*n*, %)**	-	-	-	-

C1-INH-HAE: hereditary angioedema due to C1-esterase inhibitor deficiency; FXII-HAE: hereditary angioedema due to mutations in factor XII; MYOF-HAE: hereditary angioedema due to mutations in factor Myoferlin gene; LTP: long-term prophylaxis, pdC1-INH: plasma-derived C1-inhibitor concentrate. * One out of two patients with FXII-HAE presented with attacks only during her pregnancies. The other patient was always asymptomatic. In fact, her diagnosis was made during a familiar genetic screening.

**Table 2:** Prevalence of the most reported adverse reactions after the first dose and the second dose in our HAE patients (*n* = 31) vs. healthy Italian subjects (*n* = 6,612). Adapted from Monami, et al. Ann. Ig. 2022, 34, 344–357 [19].

Symptom	HAE First Dose *n* (%)	Italian Subjects First Dose *n* (%)	HAE Second Dose *n* (%)	Italian Subjects Second Dose *n* (%)
**Pain, itching, paraesthesia** **(Vaccination site)**	18 (58%)	3164 (50.7%)	18 (58%)	811 (48.5%)
**Fever**	6 (19.3%)	285 (4.6%)	8 (25.8%)	275 (16.5%)
**Fatigue**	0 (0%)	1435 (23.0%)	6 (19.3%)	871 (52.1%)
**Myalgia/arthralgia**	1 (3.2%)	800 (12.8%)	8 (25.8%)	797 (47.7%)
**Headache**	4 (12.9%)	1148 (18.4%)	7 (22.5%)	650 (38.9%)
**Nausea and/or vomiting**	2 (6.4%)	111 (1.8%)	2 (6.4%)	34 (2.0%)
**Diarrhea**	1 (3.2%)	138 (2.2%)	0 (0%)	93 (5.6%)
**Limb paraesthesia**	1 (3.2%)	15 (0.2%)	0 (0%)	5 (0.3%)

## Data Availability

The data presented in this study are available on request from the corresponding author.
